# Anti-Inflammatory, Antioxidant, and Skin Regenerative Potential of Secondary Metabolites from Plants of the Brassicaceae Family: A Systematic Review of In Vitro and In Vivo Preclinical Evidence (Biological Activities Brassicaceae Skin Diseases)

**DOI:** 10.3390/antiox11071346

**Published:** 2022-07-10

**Authors:** Patricia da Silva Mattosinhos, Mariáurea Matias Sarandy, Rômulo Dias Novaes, Debora Esposito, Reggiani Vilela Gonçalves

**Affiliations:** 1 Department of General Biology, Federal University of Vicosa, Vicosa 36570-900, MG, Brazil; patricia.mattosinhos@ufv.br (P.d.S.M.); mariaurea.souza@ufv.br (M.M.S.); 2 Department of Structural Biology, Federal University of Alfenas, Alfenas 37130-001, MG, Brazil; romulo.novaes@unifal-mg.edu.br; 3 Plants for Human Health Institute, North Carolina Research Campus, NC State University, 600 Laureate Way, Kannapolis, NC 28081, USA; daesposi@ncsu.edu; 4 Department of Animal Science, NC State University, 120 Broughton Drive, Raleigh, NC 27695, USA

**Keywords:** Brassicaceae, inflammation, murine, epithelial cells, cytokines, inflammatory mediators

## Abstract

The Brassicaceae family constitutes some of the most well-studied natural products in the world, due to their anti-inflammatory, anti-oxidative, and pro-regenerative properties as well as their ubiquitous distribution across the world. To evaluate the potential efficacy of the Brassicaceae family in the treatment of inflammatory skin disorders and wounds, based on preclinical evidence from in vivo and in vitro studies. This systematic review was performed according to the PRISMA guidelines, using a structured search on the PubMed-Medline, Scopus, and Web of Science platforms. The studies included were those that used murine models and in vitro studies to investigate the effect of Brassicaceae on skin disorders. Bias analysis and methodological quality assessments were examined through SYRCLE’s RoB tool. Brassicaceae have shown positive impacts on inflammatory regulation of the skin, accelerating the wound healing process, and inhibiting the development of edema. The studies showed that the Brassicaceae family has antioxidant activity and effects on the modulation of cyclooxygenase 2 and the nuclear factor kappa β (NFκβ) pathway. The secondary metabolites present in *Brassicas* are polyphenols (68.75%; *n* = 11), terpenes/carotenoids (31.25%; *n* = 5), and glycosylates (25%; *n* = 4), which are responsible for their anti-inflammatory, healing, and antioxidant effects. In addition, the current evidence is reliable because the bias analysis showed a low risk of bias. Our review indicates that compounds derived from Brassicaceae present exceptional potential to treat inflammatory skin diseases and accelerate cutaneous wound healing. We hope that our critical analysis can help to expedite clinical research and to reduce methodological bias, thereby improving the quality of evidence in future research. The registration number on the Prospero platform is CRD42021262953.

## 1. Introduction

The skin is the largest organ of the human body and has several roles, including protection against microorganisms and thermoregulation [1]. Many alterations can affect the integrity of this organ, usually resulting in diseases [2]. Therefore, skin diseases and changes represent a serious health problem worldwide and are frequently associated with high costs for treatment and inefficient medicines [3]. The problem is due to the complexity and lack of understanding of the mechanisms involved during the inflammatory process. In each stage of skin lesion recovery, different cells and mediators act to promote tissue regeneration, independent of the etiology. The skin recovery process is separated into inflammation, proliferation, and tissue remodeling phases [4]. If the inflammation process persists, excessive release of inflammatory mediators, free radicals and reactive oxygen species (ROS) by macrophages and neutrophils occurs, promoting oxidative stress [5]. The mechanism that explains a direct relationship between inflammation and oxidative balance is known as respiratory burst, or inflammasome, in which the greater the inflammation, the greater the tissue oxidative imbalance [6]. Therefore, the control of inflammation is essential for a good recovery of the skin tissue. Therapeutic interventions that assist in rapid recovery and promote infection control and consequently the resolution of the inflammatory process are increasingly necessary.

In this sense, the search for natural products for the treatment of skin diseases has been growing. Among these products, we can highlight compounds obtained from the Brassicaceae, one of the largest plant families distributed on all continents except the Antarctic [7]. *Brassicas* are not endemic to Brazil but are the most cosmopolitan plant family in the plant kingdom. Their greatest diversity is centered in the temperate regions of the northern hemisphere, extending from the Mediterranean to Central Asia [8]. They have great importance for medicine, being used as anti-inflammatory, antioxidant, and healing agents. These properties are related to their chemical composition, especially flavonoids, folic acid, amino acids, vitamins, and minerals [9]. This family comprises several secondary metabolites (glucosinolates, e.g., sinigrin), hormones (e.g., brassinosteroids), and amino acids that contribute to its anti-inflammatory and healing properties [10,11]. In addition to its probable action and effectiveness compared to drugs, it has a low cost compared to the drugs, making it affordable for the population.

The available evidence on doses, timing, and different types of compounds obtained from *Brassicas* is rather fragmented. In this context, we used a systematic review framework to integrate preclinical evidence (in vitro and in vivo) to investigate the relevance of the use of Brassicaceae in skin disorders. In addition, the methodological quality of each in vivo study was assessed, pointing out the main sources of bias that undermine the quality of the current evidence and that should be overcome in natural options to treat skin changes.

## 2. Methods and Materials

### 2.1. Focus Question

The main question to be answered in this systematic review was: What is the influence of the Brassicaceae family on skin inflammatory diseases using in vitro and in vivo analysis?

### 2.2. Search Strategy

**Figure 1:**
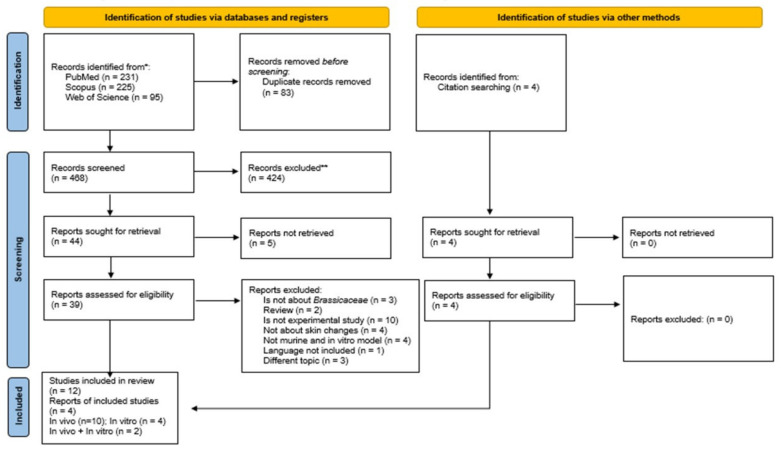
Preferred Reporting Items for Systematic Reviews and Meta-Analyses (PRISMA) flow diagram. The flowchart indicates the research records obtained at all standardized stages of the search process required for the development of systematic reviews and meta-analyses. Based on the PRISMA statement (http://www.prisma-statement.org, accessed on 25 June 2022). * Consider, if feasible to do so, reporting the number of records identified from each database or register searched (rather than the total number across all databases/registers). ** If automation tools were used, indicate how many records were excluded by a human and how many were excluded by automation tools.

This systematic review followed the Preferred Reporting Items for Systematic Reviews and Meta-Analyses (PRISMA) guidelines [12] (Figure 1), which were used as a guide for study selection, screening, and eligibility. Based on two parameters, a search strategy was developed to maximize the retrieval of relevant study registers. The first parameter was based on a direct advanced search in the electronic databases PubMed/Medline (www.ncbi.nlm.nih.gov/pubmed, accessed on 12 April 2021), Scopus (www.scopus.com, accessed on 12 April 2021), and Web of Science (www.webofknowledge.com, accessed on 12 April 2021). In the second parameter, an indirect screening was performed by carefully reading the reference list of selected articles previously in the databases, to select potential studies to be included in the systematic review. For all databases, search filters were based on: (i) skin and (ii) Brassicaceae.

A search filter was initially developed for PubMed/Medline according to standardized descriptors (MeSH terms) organized in the hierarchical tree of the MeSH database (www.ncbi.nlm.nih.gov/mesh, accessed on 10 April 2021). The commands (MeSH Terms) and TIAB were combined to broaden the retrieval of relevant indexed studies and those in the indexing process. The search matrix used in the PubMed/Medline database was adapted for Scopus and Web of Science by using the search algorithms TITLE-ABS-KEY or TS=, respectively [13]. Table S1 fully describes the search strategy for this review.

Two reviewers (PSM and MMS) conducted the literature search, removed duplicate articles, and examined titles and abstracts according to eligibility criteria. In case of discrepancies, another group of reviewers (RDN, DAE, and RVG) decided whether the study met the inclusion criteria.

Two independent researchers (PSM and MMS) conducted the literature search. The kappa test was performed for data selection (kappa = 0.911).

### 2.3. Eligibility Criteria

After initial screening and removal of duplicates, the full text of potentially relevant studies was retrieved and then assessed for eligibility for inclusion in the review. Study exclusion was based on well-defined criteria as follows: (i) studies ex vivo, (ii) studies with humans; (iii) secondary studies (i.e., editorials, commentaries, letters to the editor, literature reviews without original data). In addition, no language limits were applied in the primary search. All studies published up to April 2021 were included in the systematic review. The search strategy is detailed in the supplementary materials (Table S1). Only studies that met the following eligibility criteria were selected:

(1) Studies that evaluate *Brassicas* exposure in the skin changes.
(2) In vivo and in vitro studies of changes in the skin with murine models.
(3) Studies evaluating the effect of Brassicaceae on the inflammatory process associated with edema and wound healing.

### 2.4. Data Extraction and Synthesis from In Vivo and In Vitro Studies

The essential data from each study were extracted through structured tables according to the following descriptive levels: (i) Publication characteristics (authors, years, and countries); (ii) Characteristics of the experimental model (species, sex, age, weight); (iii) Characteristics of the target organ: normal skin or skin with changes (wound healing; contact dermatitis; edema); (iv) Information about *Brassica* treatment (species of plant used, dose, frequency of administration, pharmaceutical form, and treatment in the control group); (v) Primary outcomes (skin change, cellular analysis, vascularization, extracellular matrix analysis, molecular analysis (cytokine and growth factor analyses), epithelialization rate and/or wound closure); (vi) Secondary outcomes (oxidative stress analyses, wound tensile strength, epidermis thickness).

The characteristics described in the descriptive levels (i), (iv), (v), and (vi) were also extracted and analyzed from in vitro studies. Other characteristics were also evaluated for the in vitro studies, such as cell type, cell lineage, culture medium, and incubation/treatment time.

### 2.5. Risk of Bias Assessment from In Vivo Studies

The risk of bias was analyzed using the Systematic Review Centre for Laboratory animal Experimentation (SYRCLE) tool. This instrument is based on the Cochrane Collaboration’s tool for assessing the risk of bias in randomized trials and is adjusted for aspects of bias that play a specific role in animal intervention studies. To increase transparency and applicability, standardized signaling questions were applied as a way to guide the judgment of researchers, based on the following domains: (i) sequence generation, (ii) allocation concealment, (iii) blinding of participants and personnel, (iv) blinding of outcome assessment, (v) incomplete outcome data, (vi) selective reporting, (vii) animal information, (viii) intervention, (ix) ethical approval, (x) tool validated, (xi) statistical methods, (xii) applicability and (xiii) other bias. The items in the RoB tool were scored with “low risk of bias”; “high risk of bias”; or “unclear” (indicating that the item was not reported, and, therefore, the risk of bias was unknown). Adherence to individual quality criteria obtained in the SYRCLE system was expressed graphically using the Review Manager 5.3 program (The Nordic Cochrane Centre, Copenhagen, Denmark, The Cochrane Collaboration). Other characteristics were also evaluated for the in vitro studies, such as cell type, cell lineage, culture medium, and incubation/treatment time.

## 3. Results

### 3.1. PRISMA Guideline

From the PubMed/MEDLINE, Scopus, and Web of Science databases, 551 articles were recovered. A total of 83 studies were duplicated, and 424 with inadequate thematic were excluded after reading the title and abstract. Of the 44 remaining studies, 32 articles were excluded after reading the full text for not meeting the eligibility criteria. Therefore, 12 studies were included in the systematic review. The reference list of all included studies was analyzed to ensure the identification of additional relevant studies, and four of them were recovered, totaling 16 studies, with 11 articles from in vivo studies, four articles from in vitro studies, and two from both. The flowchart and each step performed in the selection process to retrieve relevant studies are shown in Figure 1.

### 3.2. Characteristics of Publication

**Figure 2:**
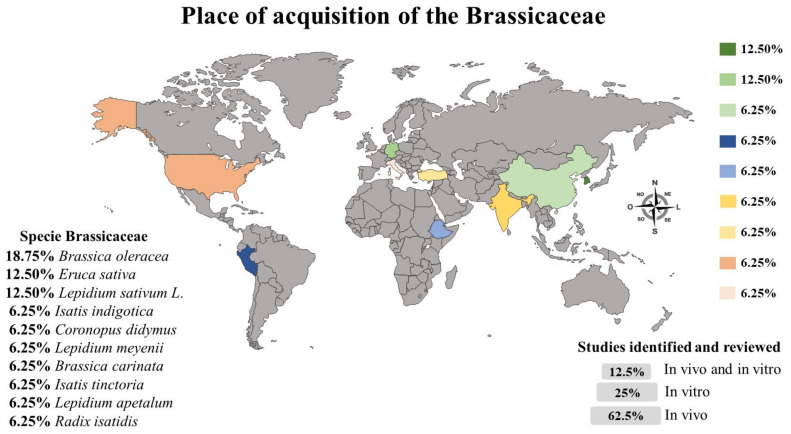
The places of acquisition of the *Brassicas* used in the studies are represented in Figure 2, as well as the types of studies (in vitro and/or in vivo) developed and the Brassicaceae species studied. The place of acquisition was reported in 31.25% of the studies reviewed, and the remaining studies did not report this information; 12% of the studies did not report the species studied.

Among the studies included in this review, 62.5% of the studies evaluated the effect of *Brassica* in animal models (*n* = 10), 25% in vitro (*n* = 4), and 12.5% used both (*n* = 2). Regarding the locality of the studies in this review, Brazil was the predominant country with 33.33% of the studies on Brassicaceae (*n* = 4), followed by India, Ethiopia, Peru, Taiwan, Korea, Spain, and the United States of America (USA), which had the same proportion of studies (8.33%; *n* = 1). Only one study did not report this information (8.33%; *n* = 1). The species and origin (where they were obtained) of *Brassicas* were reported in 62.5% of the studies (*n* = 10), in 25% of the studies only the species (*n* = 4), in 6.25% of the studies only the place of acquisition of the plant (*n* = 1), and one study reported neither the species nor origin (6.25%; *n* = 1). Figure 2 presents the *Brassicas* acquisition sites and the proportion of in vivo and in vitro studies included in this review, as well as the species studied.

### 3.3. In Vivo Model

#### 3.3.1. Characteristics of the Animal Model

The most used animal models were rats (58.33%; *n* = 7), followed by mice (41.67 *n* = 5). Among the rats, the predominant strain was Wistar in 50% of the studies. Among the mice, the predominant strain was Swiss (25%; *n* = 3), followed by Balb/C (8.33%; *n* = 1) and ICR (8.33%; *n* = 1). One study reported only the rat model but did not report the strain studied. Most of studies used only males (58.33%; *n* = 7), 16.67% used only females (*n* = 2), 16.67% used both (*n* = 2), and 8.33% of the studies did not report this information (*n* = 1). The age of the animals ranged from 6 to 10 weeks (50%; *n* = 6), and this information was not reported in 50% of the studies (*n* = 6). Animal weight ranged from 20 to 250 g in 66.67% of the studies (*n* = 8), and from 280 to 319 g in 25% (*n* = 3), and this information was not reported in two studies (16.67%; *n* = 2) (Table S2) [14,15,16,17,18,19,20,21,22,23,24,25].

#### 3.3.2. Characteristics of Treatments

The topical route was more commonly used (50%; *n* = 8), followed by oral (18.75%; *n* = 3) and intraperitoneal 6.25% (*n* = 1). The duration of the intervention varied between the healing studies and the edema/inflammation induction studies. In the studies evaluating the effects of *Brassica* on wound healing, the range was 3 to 9 days (40% of studies), or from 16 to 20 days (60% of studies). For edema/inflammation, the range was 1 day (71.42% of studies) and 3 days in two studies (28.57%). Among the Brassicaceae species studied in in vivo studies, we found *Brassica oleracea* in 25% of the studies (*n* = 3), *Coronopus didymus*, *Isatis indigotica*, *Lepidium meyenii*, *Lepidium sativum* L., *Brassica carinata* and *Isatis tinctoria* in 8.33% of the studies each (*n* = 1). All of the studies used a control group, most of them using saline solution (41.67%; *n* = 5), and others (58.33%; *n* = 7).

The best results for the wound healing process (45.45%; *n* = 5), were obtained in the 10% dosage on day 16 (*n* = 4) after continuous Brassicaceae exposure. Excisional wounds were used in 80% of the studies, and one study performed both incisional and excisional wounds. Among excisional wounds, those of size 12 mm in diameter (40%) were more commonly described by the studies (Table S6). The best results were found for the extracellular matrix, especially with an increase in collagen fiber synthesis and cell quantity, especially fibroblasts. Indicating that *Brassica* exposure was important to promote the repair process and restore tissue homeostasis.

Regarding the articles that analyzed the effect of *Brassicas* on edema, five studies induced edema in the paws of animals with carrageenan (71.42%), and one study performed only the ear edema induction test with 12-O-tetradecanoylphorbol-13-acetate (TPA). Of these, in addition to the induction of edema using carrageenan, they also analyzed the effects of *Brassica* with other tests, such as formalin (*n* = 1), TPA (*n* = 1) and with other irritating agents such as croton oil, arachidonic acid, capsaicin, and indomethacin, which were all tested in a single study (Table S6). In consideration of the induction of edema by the carrageenan test, there were large variations in the doses and time durations of *Brassica* exposure (125 to 1500 mg/kg and 1 h to 48 h). Additionally, the best results were observed after exposure to high doses in a short period (1 h), unlike low doses that needed a longer period of exposure (equal to or greater than 3 h) to show the same effect. Similarly, to all studies, at the 3 h time point (100%; *n* = 6) *Brassica* acted positively, regardless of the large dose variation, inhibiting the development of edema in the animals’ paws. As for the study that induced edema by TPA, the dose used in the intervention was 5 mg/kg (extremely low dose); it also needed a longer exposure time to show the result of edema reduction (Tables S4 and S5).

#### 3.3.3. Main Outcomes

In the healing studies, we observed that *Brassica* increased wound strength due to increased collagen type I fibers (50%; *n* = 3) and the formation of granulation tissue rich in new blood vessels. In addition, there was an improvement in the nutrition of new tissue (50%; *n* = 3), providing an increase in re-epithelialization in a short period (33.33%; *n* = 2) and increasing the number of inflammatory cells in the acute phase (e.g., macrophages) (50%; *n* = 3). Most of the papers included in this review justified these effects with the antioxidant (33.33%; *n* = 2) and anti-inflammatory (*n* = 7) capacity of the Brassicaceae. Among the antioxidant effects were reduction of free radicals and ROS and the decrease in the activity of antioxidant enzymes. Like anti-inflammatory potential, we can highlight macrophage activation inhibition and downregulation of inflammatory mediators such as nitric oxide, prostaglandin 2 (PGE2), and inflammatory cytokines (TNF-α and IL-6) (50%; *n* = 3). Brassicaceae exposure also inhibited the cyclooxygenase 2 (COX2) pathway (67%; *n* = 1) and the phosphorylation of Iκβα, preventing the translocation of NFκβ (33.33%; *n* = 2) to the nucleus and consequently preventing the gene expression of inflammatory mediators. Possibly the Brassicaceae exposure positively interferes in many other inflammatory and proliferative pathways; however, the number of papers that described this was small, and sometimes the results were fragmented. These findings demonstrated the importance of focusing studies in this area.

#### 3.3.4. Reporting Bias In Vivo Studies

**Figure 3:**
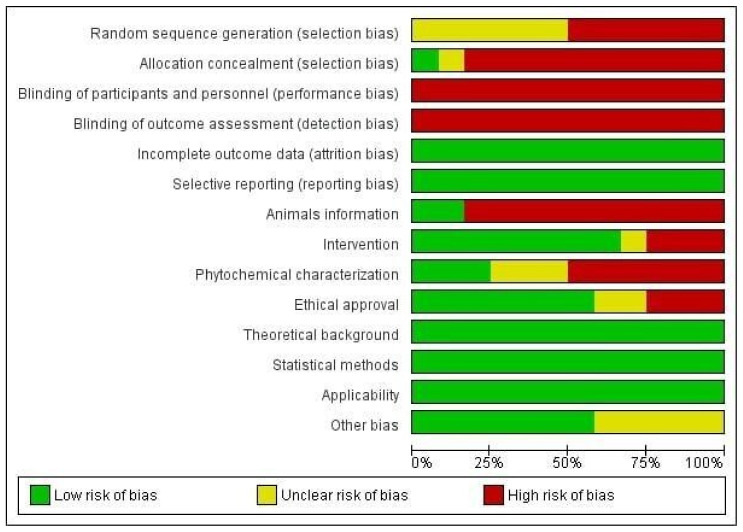
Results for the risk of bias and methodological quality indicators for all in vivo studies included in this systematic review that evaluated the effects of the Brassicaceae family on skin changes. The items in the Systematic Review Centre for Laboratory animal Experimentation (SYRCLE) Risk of Bias assessment were scored with “yes”, indicating low risk of bias (green), “no”, indicating high risk of bias (red), or “unclear”, indicating that the item was not reported, resulting in an unknown risk of bias (yellow).

**Figure 4:**
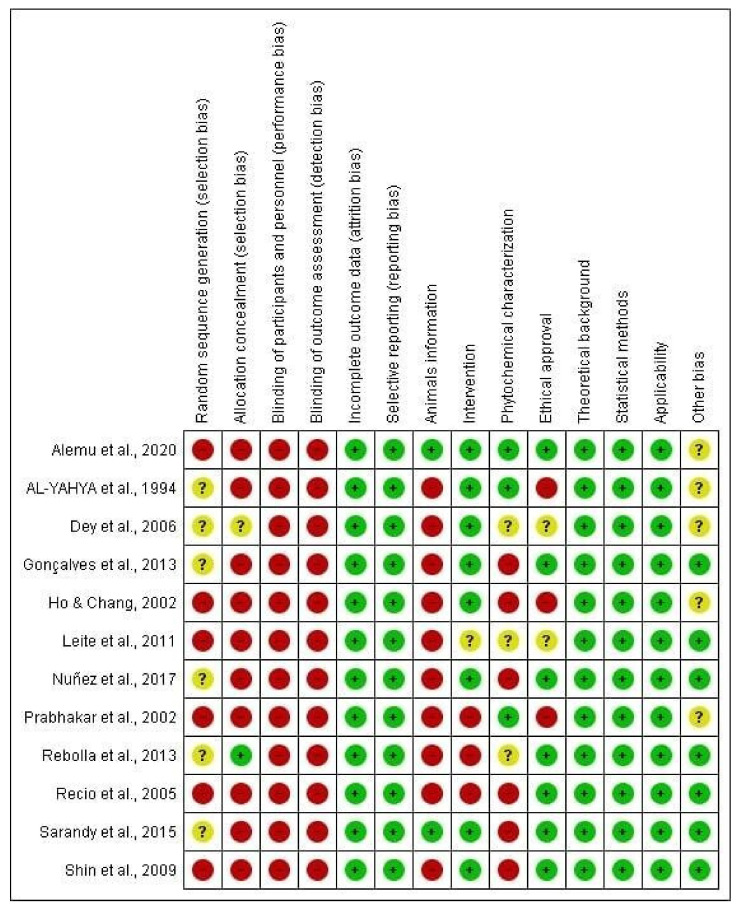
Risk of bias summary: review authors’ judgments about the risk of bias items for each included study. Green: low risk of bias; yellow: unclear risk of bias; and red: high risk of bias. References of the articles in the figure: [14,15,16,17,18,19,20,21,22,23,24,25].

The detailed results for the analysis of bias using SYRCLE’s tool are shown in Figure 3 and Figure 4. Five items were fully described in all studies (100%): namely, incomplete outcome data, selective reporting, theoretical background, statistical methods, and applicability. All of the previous items were evaluated with a low risk of bias (100%; *n* = 12). Referring to the sequence of assessed items described in Figure 3 and Figure 4, the following items were predominantly assessed with a low risk of bias: characteristics of the intervention (66.66%; *n* = 8), ethical approval on the use of experimental animals (66.66%; *n* = 8) and other bias (presentation of images, tables, and graphs) (50%; *n* = 6), in addition to the items already described above. Four items were evaluated as “unclear”, i.e., with incomplete information since the data were presented in the study, but in a way that was unsatisfactory for an understanding, such as random sequence generation (50%; *n* = 6), allocation concealment (8.33%; *n* = 1), ethical approval (16.67%; *n* = 2), and other bias (presentation of images, tables, and graphs) (41.66; *n* = 5). Finally, the items that were predominantly assessed with a high risk of bias included allocation concealment (83.33%; *n* = 10), blinding of participants and personnel (100%; *n* = 12), blinding of outcome assessment (100%; *n* = 12), and animal characteristics (83.33%; *n* = 10). However, some items were needed to improve the reports by reporting more accurate information and presenting images, tables, and graphs that facilitate the reader’s understanding of the discussed topic. The methodological quality of the studies included in this review was predominantly classified as low risk, indicating that most of the features needed for biased study evaluation were sufficiently reported. Few studies were evaluated with incomplete information. Incomplete characterization of animal models, acquisition of the species studied, treatment protocols, and especially phytochemical characterization of the plant contributed to the increased risk of bias. In addition, most studies did not report on the randomization of animals or on the blinding of animals and caregivers, which ended up generating bias in the results.

### 3.4. In vitro Models

#### 3.4.1. Characteristics of the Culture Cells

Most in vitro studies investigated the effects of Brassicaceae on human keratinocytes (50%; *n* = 3), especially the HaCaT lineage (66.67%; *n* = 4). Monocytes/macrophages and fibroblasts were also used (33.33%; *n* = 2, and 16.67%; *n* = 1, respectively). Most cells were cultured in DMEM (83.33%; *n* = 5) or EPI-500 (16.67%; *n* = 1) medium. The main characteristics related to in vitro studies are described in detail in Table S3 [16,25,26,27,28,29].

#### 3.4.2. Characteristics of Treatments

Four studies (26.67%) investigated the effects of Brassicaceae from in vitro models, and two studies investigated in vivo and in vitro models (13.33%). The duration of the in vitro study interventions varied (from 24 to 72 h) depending on the test and analysis performed. The species more studied were *Eruca sativa* in 50% (*n* = 2) and *Lepidium sativum* in 25% (*n* = 1), and one of the studies used the compound sinigrin found in *Brassicas*. Some of the studies that were performed in vivo and in vitro used the species *Lepidium apetalum* and *Radix isatidis*. Most of the studies that performed cell stimulation used lipopolysaccharide (LPS) (50%; *n* = 3), which is an integral component of the outer membrane of Gram-negative bacteria. The ranges of doses administered were 1 μM to 5 μM (16.67%; *n* = 1) and 0 μg/mL to 500 μg/mL (83.33%; *n* = 5). The characteristics and treatment protocols related to Brassicaceae are described in Table S5.

#### 3.4.3. Main Outcomes

**Figure 5:**
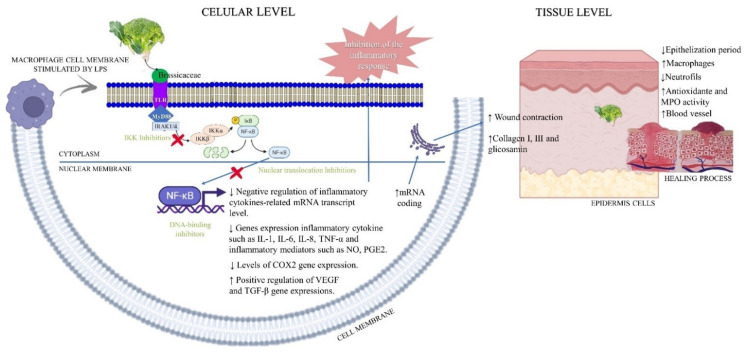
A summary of the results of the studies included in this systematic review on Brassicaceae applied in the treatment of skin alterations. In vivo and in vitro results are shown merged in the representation.

In general, the results obtained from in vitro studies indicated that *Brassica* could control positively pro-inflammatory markers such as TNF-α (66.67%), IL-1β (50%), COX2 (33.33%), IL-6 (33.33%), and nitric oxide synthase (33.33%; *n* = 2), promoting a reduction in mRNA transcription and controlling the first step of skin repair. Two other studies evaluated the proliferation and viability of RAW 264.7 macrophage cells (33.33%), and one study performed the in vitro experiments with fibroblasts (16.67%); both did not stimulate the cell migration. Brassicaceae exposure promoted a decrease in LPS-induced phosphorylation of ERK in a dose-dependent manner, while it did not affect the phosphorylation of p38 or JNK (16.67%; *n* = 1). Furthermore, the treatment significantly reduced the recruitment of myeloid differentiation factor 88 (MyD88), preventing the phosphorylation of Iκβα (33.33%; *n* = 2) and consequently the translocation of NFκβ to the nucleus, decreasing the gene expression of pro-inflammatory mediators. On the other hand, *Brassicas* also influenced the gene expression of anti-inflammatory growth factors, up-regulating the gene expressions that are important to resolve the inflammatory process. Such as vascular endothelial growth factor (VEGF) (33.33%; *n* = 2) and transforming growth factor-beta (TGF-β) (16.67%; *n* = 1), which play a role in the production of new blood vessels and the control of proliferation, respectively—events that have been described previously (Figure 5).

### 3.5. Secondary Metabolites Found in Brassicas

**Figure 6:**
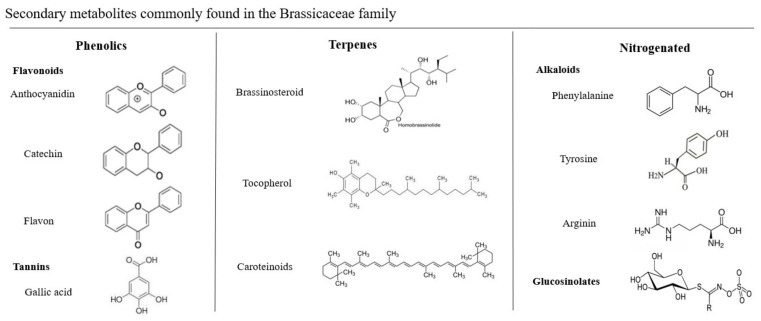
Secondary metabolites commonly found in the Brassicaceae family: phenolics, terpenes, and nitrogenated.

Our results show us that although *Brassicas* belong to the same family, these species show great diversity in terms of their metabolites composition, both quantitatively and qualitatively. Studies of secondary metabolites have developed rapidly in recent decades, due to their extensive biological activities in medicine, being an important source of pharmacologically active substances. In addition, they are known to play an important role in the adaptation of plants to their environments (abiotic and biotic stresses) and present nutritional value for human health. An analysis of the articles in this review showed that the positive effect of cruciferous vegetables on the inflammatory process during wound healing and edema development may be associated with the presence of health-promoting phytochemicals, such as polyphenols (68.75%; *n* = 11), terpenes/carotenoids (31.25%; *n* = 5), and glucosinolates (25%; *n* = 4). Some studies analyzed the effects of *Brassica*, but did not perform a phytochemical characterization and did not report on the metabolites present in the chosen species (31.25%; *n* = 5). Figure 6 shows in detail the chemical structures of the compounds commonly found in Brassicaceae.

## 4. Discussion

### 4.1. General Characteristics

Our findings indicated that the effects of these plants on the skin inflammatory process have been investigated since 1994 in different countries. Most studies were predominantly conducted in Brazil, followed by India, Ethiopia, Peru, Taiwan, Korea, Spain, and the USA. Many classes of active compounds have been isolated from Brazilian medicinal plants [30]. Brazil has the highest total biodiversity in the world and is traditionally known for the use of medicinal plants for the treatment of different acute and chronic diseases [31]. Therefore, it is easy to understand the focus on Brazil when thinking about the development of new compounds obtained from Brassicaceae. In Brazil, there is an important government organization, National Health Surveillance Agency (ANVISA), that controls studies with natural compounds. Understanding the action of the compound, its properties, and its safety for use by the population is of utmost importance to researchers for future formulations of natural bioactives. In most studies, Brassicaceae species originated from Korea and Germany, later in China, Peru, Ethiopia, India, Turkey, the USA, and Italy. Most of the plants chosen by the studies are traditionally used by the people, which may have been the predominant factor that attracted the researchers’ attention. Brassicaceae are considered important food crops in China, Japan, India, and European countries [32,33].

### 4.2. Characteristics of In Vivo Studies

Murine excisional wound models were the primary animal model used in the studies included in this systematic review about the effects of Brassicaceae on skin edema and wounds (rats and mice, respectively). This is probably because rats and mice have long been the preference for animal models in research in various areas, due to their anatomical, physiological, and especially genetic similarities with humans [34]. Another important factor is that the greater the uniformity of the animals concerning experimental, environmental, and genetic variables, the smaller the sample size of animals needed to develop the research [35]. These animal models afford low acquisition costs easy handling and can be used in large quantities in experiments, providing a higher degree of reliability in the results. Moreover, these models allow macroscopic, biochemical, and biomechanical measurements and the monitoring of the different phases of the wound healing process, especially the inflammatory and proliferative phases [36]. Male animals were predominantly used to the detriment of females. Possibly, this choice is due to different behaviors associated with the hormonal cycles of females, which can compromise the results of experiments. In addition, females are more sensitive to acute oral toxicity, as well as dermal toxicity, than males [14]. The age groups of the animals were similar, which is an important feature for studies evaluating wound healing and even intrinsically in studies of the inflammatory process, since aging rats influence the formation of new vessels, wound closure, re-epithelialization, and the resolution of the inflammatory process [34], due to the activity of enzymes that break down with aging [37]. Excisional wounds were predominant, although the number and size of wounds were variable. Possibly, the excisional wound model was chosen due to the wound closure time and to permit the best understanding of all phases of tissue repair, with a focus on the inflammatory, proliferative, and remodeling phases [38]. Besides this, the number of wounds is important information that implies the therapeutic outcome and can compromise the quality of evidence. The number of wounds produced on the animals was very heterogeneous, with two, three, or five wounds on each animal. Ideally, only one wound per animal should be produced to avoid the stress and reduce bias in the results, and consequently to reduce cytokines and growth factors that can act negatively on wound repair [39,40].

The use of skin irritants has been more common to promote tissue inflammation and edema. Mouse paw edema has been increasingly used to test new anti-inflammatory drugs, as well as to understand the mechanisms involved in inflammation [41]. Carrageenan-induced edema is a widely used test to establish anti-inflammatory activity and is a routine simple animal model for assessing pain at the site of inflammation [42,43]. TPA is an active constituent of croton oil that induces topical inflammation and a hyperproliferative response in animals in a manner like many skin diseases and in the development of edema [44]. It is often used to activate the signal transduction enzyme, protein kinase C (PKC), which is involved in the regulation of epidermal keratinocyte growth and differentiation [45]. Studies have revealed that seeds of *Sinapis alba* L. or *Brassica juncea* (L.) Czern. et Coss. are effective anti-inflammatory agents against acute and chronic inflammatory processes, an effect possibly mediated by suppressing mRNA expression of inflammatory mediators including TNF-α, IL-6, and IL-1β [46].

The most used route of administration was topical, followed by oral and intraperitoneal. These findings can be justified in the edema studies by the great local effect that is promoted by topical administration directly to the site of inflammation, permitting direct contact with the compounds responsible for anti-inflammatory activity [47]. Aspects of dosimetry depend on the biological effect and other factors of each molecule. Thus, although medicinal effects are influenced by dose and treatment time, generalizations cannot be made for molecules with different biological properties [39].

In general, the animals that received *Brassica* at 10% doses showed an increase in the rate of wound contraction, an increase in collagen fibers, and an increase in cellularity, leading to faster wound healing. In the edema studies, extracts of *Brassica* at doses of 125 mg/kg to 1500 mg/kg inhibited the development of edema in murine models, thus acting as an optimal anti-inflammatory. Ref. [48], studying the effect of some flavonoids in experimental models of inflammation in rats, demonstrated significant inhibition of joint edema induced by carrageenan with a prior intraperitoneal injection of quercetin at doses of 50 mg/Kg and 75 mg/Kg, respectively. Interestingly, most of the studies in this review reported dose-dependent results, varying according to the Brassicaceae species studied.

The studies on this theme were strong; however, it is evident that some studies were weak due to a lack of information that may have generated difficulty in reproducibility and understanding by other researchers. The risk of bias revealed specific limitations in the research reports, associated with underreporting of information, such as random sequence generation, random evaluation of results, and blinding of participants. Objectively, the limitations cited above do not indicate that the researchers did not evaluate these parameters, but it is strong evidence that this information was not included in the research reports. Therefore, considering these limitations, we hope that this systematic review, while admitting its intrinsic qualitative nature in describing important points of bias, will contribute to future studies by reporting elements of bias.

### 4.3. Characteristics of In Vitro Studies

The most used cells were keratinocytes, followed by fibroblasts. HaCaT cells are a lineage that is widely used in scientific research to study epidermal homeostasis and its pathophysiology, as well as to evaluate the molecular, biochemical, cellular, and histochemical behavior of normal and pathological tissues [49]. Recent studies in wound biology have clarified the molecular pathways governing the re-epithelialization of keratinocytes at wound sites, where culturing keratinocytes has enabled several studies on the healing and inflammatory process. [50]. Fibroblast cells are also widely used since they serve to analyze biochemical behavior and the action of growth factors on lesion areas [51,52]. To maintain the structure and proper function of the skin, keratinocytes and fibroblasts act synergistically with defense cells, regulating the cutaneous immune response to biological, physical, and chemical agents. Keratinocytes form the skin barrier through a highly complex differentiation process, and fibroblasts provide physiologically relevant cytokines and growth factors [53]. All of these positive points, together with the fact that these cells are easy to grow in vitro and are intrinsically related to skin disorders, make the culture of these cells an important tool to understand the effect of *Brassicas* on specific skin lesions.

### 4.4. Phytochemical Composition of Brassicaceae and Their Anti-Inflammatory, Antioxidant, and Healing Actions

Inflammation is a protective response of the body characterized by a series of reactions, including vasodilation and recruitment of immune cells and proteins to the injured site, which act by removing injurious stimuli and initiating the healing process [54]. Normally, inflammation is beneficial and efficient, but its persistence can cause excessive tissue damage, contributing to the development of inflammatory disease [55]. After tissue injury, receptors are activated, such as toll-like receptors (TLR), which recognize specific molecules and recruit proteins that activate different transcription factors, the most activated in this process being NFκβ [44]. Activation of NFκβ is accomplished by inducible degradation of Iκβα triggered through its phosphorylation by the Iκβ kinase (IKK) complex [56]. Upon activation, IKK phosphorylates Iκβα, triggering the degradation of Iκβα in the proteasome, resulting in the translocation of NFκβ to the nucleus [55]. When NFκβ is in the nucleus, it initiates the transcription of various inflammatory mediators and pro-inflammatory cytokines, such as TNF-α, IL-1, and IL-6, which can be overproduced. Anti-inflammatory properties of Brassicaceae have been attributed to an inhibitory effect on inflammatory cell infiltration [57], delay of pro-inflammatory gene expression, and reduction in the biosynthesis of cytokines such as TNF, IL-1, IL-6 [58]. This in vivo effect was comparable to that of the nonsteroidal anti-inflammatory aspirin, which has the property of antiplatelet aggregation. Among the secondary metabolites found in *Brassicas* that can be associated with these therapeutic effects are phenolic compounds, especially flavonoids, with quercetin and kaempferol being the predominant flavonoids in kale [59]. Flavonoids are aromatic substances containing 15 carbon atoms (C15) in their basic skeleton. This large number of compounds arises from the wide variety of combinations of methyl and hydroxyl groups as substituents in the basic chemical structure. Among the flavonoids, anthocyanins stand out, which are glycosides that present in their chemical structure a sugar residue at carbon 3 [60]. The flavonoids present in Brassicaceae (such as quercetin and catechins) act by modulating the cells involved in inflammation, inhibiting the production of TNF-α and IL-1, modulating the activity of enzymes such as COX2, and modulating the nitric oxide-forming enzyme [61,62]. Their main mechanism of action is the inhibition of COX, which converts arachidonic acid into prostaglandins [63]. Another important class of phenolic compounds found in *Brassicas* are tannins, and their mechanisms of action are related to three properties: complexation with metal ions (such as iron, copper, and calcium), antioxidant and free radical scavenger activity, and the ability to compete with macromolecules, such as proteins and polysaccharides [64,65]. In the human organism, they act as an antioxidant, antiseptic, cicatrizing agent, and vasoconstrictor [60]. Tannins serve as the basis for many structures with diverse functions in specialized and primary metabolism, ranging from rather small, volatile molecules (e.g., mono- and sesquiterpenes) to hormones (such as brassinosteroids and abscisic acid) and structural cellular components such as carotenoid pigments [66,67]. The group of nitrogen compounds includes the alkaloids, cyanogenic glycosides, and non-protein amino acids. Alkaloids are the main nitrogen compounds in plant species and are formed of different structures and composed of amino acids such as tyrosine, phenylalanine, tryptophan/tryptamine, ornithine/arginine, and histidine [68]. Included in the nitrogen compounds are also glucosinolates (GSLs), which are broken down and release hydrocyanic acid (HCN) when the plant suffers some kind of injury [69,70]. The anticarcinogenic effects of GSLs are the most commonly mentioned, but recent studies have found other beneficial activities of GSLs, including regulatory functions in the inflammatory response and antioxidant activities [71]. The biological activities of GSLs can be attributed to their hydrolytic products, of which isothiocyanates (ITCs) are prominent examples. ITCs may act as modulators of inflammation because they can reduce or inhibit the activity of the nuclear factor “kappa-light- chain-enhancer” activated β-cells (NFκβ) [72]. NFκβ regulates the expression of cyclooxygenase 2 (COX-2), which is responsible for elevated prostaglandin levels and a key inducer of inflammatory processes. Ref. [73] demonstrated that TNF-α secretion was inhibited at a concentration of 1 μM in the presence of GSLs. In addition, cytokines themselves can lead to ROS formation, which establishes a vicious circle between oxidative stress and the generation of pro-inflammatory cytokines [74,75]. Part of this effect comes from the modulation of the signaling pathway of transcription factors involved in the pro-inflammatory and anti-inflammatory pathways. *Brassicas* acted as a good antioxidant, decreasing levels of myeloperoxidase (MPO) activity and inhibiting the expression of enzymes involved in inflammation [76]. Studies have already reported the antioxidant effects of *Brassicas* [77]; for example, broccoli (*Brassica oleracea*) extracts protect against ROS, possibly due to the presence of vitamin C, quercetin, kaempferol, lutein, tocopherol, and β-carotene [78,79,80]. The main carotenoids found in *Brassicas* are lutein, β-carotene, violaxanthin, and neoxanthin [81], but also the presence of 13-cis-β-carotene, α-carotene, 9-cis-β-carotene, and lycopene, which are involved in the antioxidant effects of these species, has been reported [82]. Ref. [83] analyzed the secondary metabolites present in *Brassicas*, which demonstrated antioxidant activity, aiding in protection against free radicals and reactive oxygen species (ROS), and thus in the prevention of chronic diseases. As for its healing action, *Brassica* demonstrated increased wound contraction and rapid re-epithelialization, which may be associated with the synergistic effects of its high polyphenol and flavonoid content. This finding is fortified by previous review studies on bioactive phytochemicals of the species *Brassica* var. *B. nigra*, *B. juncea*, *B. oleracea*, and *B. rapa* [14].

The registration number on the Prospero platform is CRD42021262953.

## 5. Conclusions

Overall, we have shown that the evidence on the healing and anti-inflammatory potential of Brassicaceae is based on valid and realistic pre-clinical models that share similar stages of tissue repair with those observed in humans and in in vitro assays. From studies using these models, we determined that Brassicaceae are potentially effective in accelerating skin wound healing and efficient in reducing edema by inhibiting the inflammatory process. Their beneficial effects are mainly associated with stimulation of wound contraction, re-epithelialization, collagenogenesis, and inhibition of the expression of inflammatory genes. All of these therapeutic effects are attributed to flavonoids, tannins, terpene, carotenoids and glucosinolates. Low doses of *Brassica* are effective in closing wounds and reducing edema in murine animals. However, a longer time exposure is required when compared to high doses of this compound, which require low time exposure to show their effects. Brassicaceae acted mainly on the NFκβ pathway, modulating the expression of inflammatory mediators and cytokines. Since most inflammatory cytokines activate NFκβ, drugs that inhibit the activation pathways of this factor may have broad-spectrum anti-inflammatory effects, as Brassicaceae have shown, expanding new perspectives for the treatment of inflammatory diseases.

## Data Availability

The data are contained within the article and Supplementary Materials.

## Supplementary Materials

The following supporting information can be downloaded at: https://www.mdpi.com/article/10.3390/antiox11071346/s1, Table S1: Detailed search strategy with search filters and the number of studies recovered in all electronic databases; Table S2. Characteristics of the experimental models used in all in vivo studies included in this systematic review; Table S3. Characteristics of all in vitro studies included in this systematic review; Table S4. Characteristics of treatments administered in all in vivo studies that were identified in this systematic review; Table S5. Characteristics of treatments administered in all in vitro studies that were identified in this systematic review; Table S6. Characteristics of skin changes from in vivo studies identified in the systematic review.
