# Supplementary material for: Anti-Inflammatory, Antioxidant, and Skin Regenerative Potential of Secondary Metabolites from Plants of the Brassicaceae Family: A Systematic Review of In Vitro and In Vivo Preclinical Evidence (Biological Activities Brassicaceae Skin Diseases)
Source: Antioxidants (Basel). 2022 Jul 10;11(7):1346. doi: 10.3390/antiox11071346 (PMC9312357; doi:10.3390/antiox11071346)
Supplement: Supplementary file 1 [file antioxidants-11-01346-s001.zip › antioxidants-1744864-supplementary.pdf]

Supplementary Material

Table S1. Detailed search strategy with search filters and the number of studies recovered in all electronic databases.

Database	Descriptors	Items found	Time	Date
Pubmed	**#1** ("brassicaceae"[MeSH Terms] OR brassicaceae[TIAB])	74.725	16:34:00	12/04/2021
	**#2** (“Skin”[MeSH terms] OR “Dermis”[MeSH terms] OR “Granulation Tissue”[MeSH terms] OR “Epidermis”[MeSH terms] OR “Keratinocytes”[MeSH terms] OR “Integumentary System”[MeSH terms] OR “Dermatology”[MeSH terms] OR “Dermoscopy”[MeSH terms] OR “Wounds and Injuries”[MeSH terms] OR “Fibrosis”[MeSH terms] OR “Skin injuries”[TIAB] OR “Skin fibrosis”[TIAB] OR “Skin scars”[TIAB] OR “Skin cicatriz”[TIAB] OR “Cicatrix”[MeSH terms])	1.364.344	16:35:00	12/04/2021
	**#1 AND #2**	231	16:43:00	12/04/2021
**Database**	**Descriptors**	**Items found**	**Time**	**Date**
Scopus	**#1** (TITLE-ABS-KEY("brassicaceae"))	10.092	12:44:00	12/04/2021
	**#2** (TITLE-ABS-KEY(Skin) OR TITLE-ABS-KEY(Dermis) OR TITLE-ABS-KEY(“Granulation Tissue”) OR TITLE-ABS-KEY(Epidermis) OR TITLE-ABS-KEY(Keratinocyte*) OR TITLE-ABS-KEY(Integumentary System) OR TITLE-ABS-KEY(Dermatology) OR TITLE-ABS-KEY(Dermoscopy) OR TITLE-ABS-KEY(Skin wounds) OR TITLE-ABS-KEY(Skin injuries) OR TITLE-ABS-KEY(Skin fibrosis) OR (Skin cicatrix))	1.394.680	12:46:00	12/04/2021
	**#1 AND #2**	225	12:50:00	12/04/2021
**Database**	**Descriptors**	**Items found**	**Time**	**Date**
	**#1** TS=brassicaceae	7.342	13:11:00	12/04/2021

**Web Of Science**	**#2** TS=Skin OR TS=Dermis OR TS=Granulation Tissue OR TS=Epidermis OR TS=Keratinocyte* OR TS=Integumentary System OR TS=Dermatology OR TS=Dermoscopy OR TS=Skin wounds OR TS=Skin injuries OR TS=Skin fibrosis OR TS=Skin cicatrix	737.406	13:13:00	12/04/2021
	**#1 AND #2**	95	13:20:00	12/04/2021

Table S2. Characteristics of the experimental models used in all *in vivo* studies included in this systematic review.

**Studies *In vivo***						
**Reference**	**Country**	**Animal model**	**Strain**	**Sex**	**Age**	**Weight**
Alemu *et al*., 2020	Etiopia	Mice	Swiss	Either sex	6-8 weeks	25-30g
Al-Yahya *et al.,* 1994	?	Rat	?	Either sex	8-10 weeks	180-200g
Dey, 2005	United States of America	Rat	Wistar	Male	?	100–200 g
Gonçalves *et al*., 2013	Brazil	Rat	Wistar	Male	?	319g
Ho & Chang, 2002	Taiwan	Rat	Wistar	Male	?	150–180 g
Leite *et al.,* 2011	Brazil	Mice	Swiss	Male	?	20-25g
Nuñez *et al.,* 2017	Peru	Mice	Balb/c	Male	9 weeks	?
Prabhakar *et al*., 2002	India	Rat	Wistar	Male	?	150–250 g
Rebolla *et al*., 2013	Brazil	Rat	Wistar	?	8-9 weeks	280 g
Recio *et al*., 2005	Spain	Mice	Swiss	Female	?	25–30 g
Sarandy *et al*., 2015	Brazil	Rat	Wistar	Male	8-9 weeks	302g
Shin *et al*., 2010	Korea	Mice	ICR	Female	6 weeks	?

?, not reported.

Table S3. Characteristics of all *in vitro* studies included in this systematic review.

**Studies *In vitro***						
**Reference**	**Country/Source**	**Cells Type**	**Cells Lineage**	**Culture medium**	**Incubation time**	**Cell estimulation**
Dey, 2005	ATCC	Monocyte/macrophage	RAW 264.7	DMEM	24h; 72h	-
Kim *et al*., 2014	United States of America	Human keratinocytes; Human fibroblasts	HaCaT/ fibroblasts	EPI-500	24h	LPS
Mazumder *et al*., 2015	South Africa	Human keratinocytes	HaCaT	DMEM	24h	-
Shin *et al*., 2010	Korea	Murine macrophages	RAW 264.7	DMEM	72h	LPS
Turkoglu *et al*., 2018	United States of America	Human keratinocytes	HaCaT	DMEM	72h	-
Yehuda *et al.,* 2009	United States of America	Human keratinocytes	HaCaT	DMEM	?	LPS

?, not reported. Not, Cells were not stimulated. DMEM, Dulbecco's Modified Eagle's medium. LPS, lipopolysaccharide.

Table S4. Characteristics of treatments administered in all in vivo studies *that were* identified in this systematic review.

References	*Plant specie*	Source	Pathology in question	Route/application	Dose	Control	Duration of treatment
Alemu *et al*., 2020	*Brassica carinata*	North Western Ethiopia	Wound healing	Topical	10%	Simple ointment (negative control); nitrofurazone skin ointment (positive control).	Once a day for 9 days
Al-Yahya *et al.*, 1994	*Lepidium sativum L.*	?	Inflammatory diseases in general	Oral	500 mg/kg	Saline solution	Single day in 0, 2 and 3h
Dey, 2005	*Lepidium apetalum*	United States of America	Inflammatory diseases in general	Oral	200 mg/kg	Apricot kernel balm	Single day in 3, 5, 24 and 48 h
Gonçalves *et al.*, 2013	*Brassica oleracea*	?	Wound healing	Topical	10%	Saline solution	Once a day for 20 days
Ho & Chang, 2002	*Isatis indigotica*	China	Inflammatory diseases in general	Intraperitoneal	1500 mg/kg	Indometacina	Single day in five intervals of 30 minutes
Leite *et al.*, 2011	?	?	Inflammatory diseases in general	Topical	20μL/ear/kg	Saline solution	Single day for 6h
Nuñez *et al.*, 2017	*Lepidium meyenii*	Peru	Wound healing	Topical	0,08% (160ug/200mg)	Silver sulfadiazine	Once a day for 3 days

						cream (positive control)	
Prabhakar *et al.*, 2002	*Coronopus didymus*	India	Inflammatory diseases in general	Oral	200 mg/kg	?	Single day in 1, 2 and 3h
Rebolla *et al.*, 2013	*Brassica oleracea var. capitata*	?	Wound healing	Topical	10%	Saline solution	Twice a day for seven and 16 days
Recio *et al.*, 2005	*Isatis tinctoria*	Germany	Dermatitis	Topical	125 and 175 mg/kg	Acetone	Twice daily for 4 days
Sarandy *et al.*, 2015	*Brassica oleracea var. capitata*	?	Wound healing	Topical	10%	Saline solution	Once a day for 20 days
Shin *et al.*, 2010	*Radix Isatidis*	Korea	Inflammatory diseases in general	Topical	5 mg/ear/kg	β-actin	Every 24 h for 3 days

Table S5. Characteristics of treatments administered in all *in vitro* studies *that were* identified in this systematic review.

References	*Plant specie*	Source	Dose	Period of incubation
Dey, 2005	*Lepidium apetalum*	United States of America	50 µg/mL	9 and 21h
Kim *et al.,* 2014	*Eruca sativa*	Korea	20 µg/mL and 100 µg/mL	24h
Mazumder *et al.*, 2015	*?*	Germany	70 µg/mL and 140µg/mL	24h
Shin *et al.*, 2010	*Radix Isatidis*	Korea	0-500µg/mL	24h; 72h.
Turkoglu *et al.*, 2018	*Lepidium sativum L.*	Turkey	1:1, 1:10, 3:100, 1:100, and 1:1000	72h
Yehuda *et al.*, 2009	*Eruca sativa*	Italy	1 µg/mL	72h

?, not reported.

Table S6. Characteristics of skin changes from *in vivo* studies identified in the systematic review.

**Type of change: Wound healing**						
**Reference**	**Lesion**	**Size area of wound**	**Number of wounds**	**Region**	**Anesthetic**	**Instrument**
Rebolla *et al.*, 2013	Excisional	30mm	?	Dorsal	Ketamine and xylazine	Surgical scalpel
Alemu *et al.*, 2020	Incisional and excisional	30mm and 300mm²	?	Dorsal	Ketamine and diazepam	Forceps and surgical blades
Sarandy *et al.*, 2015	Excisional	12 mm	3	Dorsal	Ketamine and xylazine	Surgical scalpel
Nuñez *et al.,* 2017	Excisional	10mm	2	Dorsal	Sodium pentobarbital	?
Gonçalves *et al.*, 2013	Excisional	12 mm	5	Dorsal	Ketamine and xylazine	Surgical scalpel

?, not reported.

Table S6 (continuation). Characteristics of skin changes from *in vivo* studies identified in the systematic review.

**Type of change: Edema/Inflammatory**				
**Reference** (animal model)	**Edema/Inflammation Induction**	**Region**	**Time of treatment exposure**	**Edema measurement**
Al-Yahya *et al.,* 1994	Carrageenan-induced	Right hind paw	1h before the carrageenan	0, 2 and 3 h
Dey, 2005	Carrageenan-induced	Right hind paw	1h after the carrageenan	3, 5, 24 and 48h
Ho & Chang, 2002	Carrageenan-induced	Right hind paw	30min before the carrageenan	Single day of 30-min intervals up to five times
Leite *et al.,* 2011	Croton oil; Arachidonic acid; Capsaicin; Indomethacin	Ear	6h after the application of each irritant agent	30min; 1h; 6h
Prabhakar *et al*., 2002	Carrageenan-induced	Right hind paw	1h before the carrageenan	0, 1, 2 and 3 h
Recio *et al.,* 2005	Carrageenan-induced	Right hind paw	1h before the carrageenan	1, 3 and 5 h
Shin *et al*., 2010	Multiple application of TPA	Ear	1h after TPA	Every 24 h for 3 days

TPA: 12-O-tetradecanoylphorbol-13-acetate.

Filename: antioxidants-1744864-supplementary.docx
Directory: C:\Users\MDPI\Documents
Template: C:\Users\MDPI\AppData\Roaming\Microsoft\Templates\Normal.dotm
Title: Type of the Paper (Article
Subject:
Author: MDPI
Keywords:
Comments:
Creation Date: 5/13/2022 9:08:00 AM
Change Number: 5
Last Saved On: 6/25/2022 12:36:00 PM
Last Saved By: MDPI
Total Editing Time: 23 Minutes
Last Printed On: 7/7/2022 2:01:00 PM
As of Last Complete Printing
Number of Pages: 7
Number of Words: 1,134 (approx.)
Number of Characters: 6,468 (approx.)
